# Long non-coding RNA-mediated competing endogenous RNA regulatory network during flower development and color formation in *Melastoma candidum*

**DOI:** 10.3389/fpls.2023.1215044

**Published:** 2023-07-27

**Authors:** Hui Li, Wei Wang, Rui Liu, Botong Tong, Xinren Dai, Yan Lu, Yixun Yu, Seping Dai, Lin Ruan

**Affiliations:** ^1^ Department of Botany, Guangzhou Institute of Forestry and Landscape Architecture, Guangzhou, China; ^2^ College of Forestry and Landscape Architecture, South China Agricultural University, Guangzhou, China; ^3^ State Key Laboratory of Tree Genetics and Breeding, Chinese Academy of Forestry, Beijing, China; ^4^ State Key Laboratory of Tree Genetics and Breeding, Northeast Forestry University, Harbin, China; ^5^ Jiangsu Key Laboratory for the Research and Utilization of Plant Resources, Institute of Botany, Chinese Academy of Sciences, Nanjing, Jiangsu, China

**Keywords:** lncRNA, ceRNA regulatory mechanism, flower development, flower color formation, metabolites, *Melastoma candidum*

## Abstract

*M. candidum*, an evergreen shrubby flower known for its superior adaptation ability in South China, has gained increased attention in garden applications. However, scant attention has been paid to its flower development and color formation process at the non-coding RNA level. To fill this gap, we conducted a comprehensive analysis based on long non-coding RNA sequencing (lncRNA-seq), RNA-seq, small RNA sequencing (sRNA-seq), and widely targeted metabolome detection of three different flower developmental stages of *M. candidum*. After differentially expressed lncRNAs (DElncRNAs), differentially expressed mRNAs (DEmRNAs), differentially expressed microRNAs (DEmiRNAs), and differentially synthesized metabolites (DSmets) analyses between the different flower developmental stages, Gene Ontology (GO) and Kyoto Encyclopedia of Genes and Genomes (KEGG) were conducted to identify some key genes and metabolites in flavonoid, flavone, anthocyanin, carotenoid, and alkaloid-related GO terms and biosynthetic pathways. Three direct-acting models, including antisense-acting, cis-acting, and trans-acting between lncRNAs and mRNAs, were detected to illustrate the direct function of lncRNAs on target genes during flower development and color formation. Based on the competitive endogenous RNA (ceRNA) regulatory theory, we constructed a lncRNA-mediated regulatory network composed of DElncRNAs, DEmiRNAs, DEmRNAs, and DSmets to elucidate the indirect role of lncRNAs in the flower development and color formation of *M. candidum*. By utilizing correlation analyses between DERNAs and DSmets within the ceRNA regulatory network, alongside verification trials of the ceRNA regulatory mechanism, the study successfully illustrated the significance of lncRNAs in flower development and color formation process. This research provides a foundation for improving and regulating flower color at the lncRNA level in *M. candidum*, and sheds light on the potential applications of non-coding RNA in studies of flower development.

## 1 Introduction

LncRNAs are an influential class of molecules with a length of more than 200 nt (Wang and Chang, 2011). They originate from exonic, intronic, intragenic, and intergenic promoter regions, as well as 3’ and 5’ UTR enhancer sequences, and are transcripted in either a sense or antisense direction (Zhang et al., 2013). Traditionally, lncRNAs were thought to be meaningless molecules. In recent years, research has proven that lncRNAs regulate many biological processes in organisms such as gene expression adjustment (Guttman et al., 2009), post-transcription, post-translation (Crick et al., 1961), and chromosome modification (Bertone et al., 2004; Yanofsky, 2007) by combining with corresponding proteins. Unlike protein-coding genes, most lncRNAs are usually expressed at low levels and lack strong sequence conservation between species (Cabili et al., 2011; Necsulea et al., 2014). A number of studies have provided evidence that lncRNAs play a significant role in response to stress (Wunderlich et al., 2014), male sterility (Ding et al., 2012), phosphate homeostasis (Franco-Zorrilla et al., 2007), flowering time regulation (Heo and Sung, 2011), and flower and pollen development (Liu et al., 2010; Kang and Liu, 2015) in plants.

An increasing body of evidence indicating that lncRNAs may affect gene expression by either *cis*-acting on their chromosomes or *trans*-acting protein-encoding genes to carry out their functions (Wu et al., 2019). It is possible that long non-coding RNAs located upstream of a gene may associate with the promoter or other *cis*-acting elements of co-expressed genes in order to regulate gene expression at the transcriptional or post-transcriptional level. A large number of long non-coding RNAs could overlap with transcription factor binding sites, potentially preventing transcription factors from binding to the corresponding sites (de la Fuente, 2010; Chen, 2016). In some cases, lncRNAs are found to enhance the binding rate of transcription factors to nearby binding sites (de la Fuente, 2010; Yang et al., 2019). A study by Li et al. (2022) found that *cis*-acting LNC_002115 regulates hickory female floral development by influencing both *PHO2* and *SVP*. Kang and Liu (2015) identified a significant number of lncRNAs from 35 different flower and fruit tissues of diploid strawberries. They inferred a *cis* or *trans*-acting relationship between lncRNAs and their targets based on correlation analysis of lncRNAs and their target gene expression trend. In addition, they discovered that lncRNAs are not well conserved between species of plants.

As endogenous target mimics, lncRNAs can bind with miRNAs on mRNA response elements (MREs) and mitigate miRNAs’ cleavage effect on target genes by sponge-like actions (Wu et al., 2019). Due to their similar structural characteristics, lncRNAs may also be negatively regulated by miRNAs through a similar mechanism to mRNAs (Chaofeng et al., 2013). In *Arabidopsis*, a ceRNA regulatory relationship showed that lncRNA IPS1 could influence the expression level of *PHO2* by binding to miR399 (Franco-Zorrilla et al., 2007). LncRNAs, *COOLAIR* and *COLDAIR*, could repress the *FLC* gene expression through an epigenetic silencing mechanism to regulate flowering time (Heo and Sung, 2011; Kim and Sung, 2017). Compared with the wild type, overexpression of lncRNA npc48 increased the rosette diameter and leaf serration and delayed flowering time (Ben Amor et al. 2009). Besides, ceRNA regulatory relations has also been demonstrated in other plants, such as cucumber (He et al., 2020), pepper (Zuo et al., 2019), rice (Xu et al., 2016), maize (Zhu et al., 2017), and tomato (Yang et al., 2019). In rice, lncRNA osa-eTM160 attenuated the repression of osa-miR160 on *ARF18* throughout the embryonic anther by target mimicry ways (Wang et al., 2017). Fang et al. (2019) found that a rice lncRNA Ef-dc transcribed from the antisense strand of the flowering activator *SOC1* locus can positively regulate the expression of *SOC1* and balance yield with maturity duration. Wang et al. (2017) found that lncRNA osa-eTM160 could attenuate the repression of osa-miR160 on osa-*ARF18* during the early developmental stage of the anther. A genome-wide association study (GWAS) analysis and ceRNA network by Xu et al. (2021) identified the key regulatory mechanism of the LTCONS_00034157- miRNA167h- PsTPS1 in the later flowering process of *Prunus sibirica*.

Flower color is an important trait that determines the ornamental quality and landscaping application value (Zhu et al., 2019b). It is the result of pigment metabolite accumulation in the vacuoles of flower epidermal cells (Mol et al., 1998; Deng et al., 2013). Flower color is predominantly due to the production of flavonoids carotenoids or betalains. (Holton, 1995). Betalains, one type of alkaloid, are water-soluble nitrogenous pigments derived from the amino acid L-tyrosine, mainly classified as red-violet betacyanins and yellow betaxanthins. In Caryophyllales, betalains exclusively replace anthocyanin (Tanaka et al., 1998; Deng et al., 2013) to attract pollinators and seed dispersers (Sunnadeniya et al., 2016). Some researchers have demonstrated that the relevant enzymes for the production of anthocyanins are not expressed in betalains-producing plants at the biochemical level (Shimada et al., 2007; Brockington et al., 2011). However, enzymes, genes and biosynthetic pathways involved in betalain production are much less well-studied than those of flavonoids and carotenoids.

The change in color both within flowers and in isolated pigments involves a range of biochemical mechanisms. Some of the factors influencing color are temperature, co-pigments, pH, metals, sugars anthocyanin stacking, and cell shape (Bowles et al., 2006; Shoji et al., 2007). The earliest research suggested that pH, metal-complex theory, and Metalloanthocyanins could be the key factors to determine flower color. In 1913, Willstätter and Everest proposed the pH theory based on the observation of a pigment from blue cornflowers and rose, cyanin, which could display red color under acidic media and blue color under alkaline solutions (Willstätter and Everest, 1913). The metal complex theory states that anthocyanin can form complexation with metal ions such as Mg^2+^(Mitsui et al., 1959), Fe^3+^, Al^3+^(Bayer et al., 1966) Ga^3+^, In ^3+^, Co ^3+^, Mn ^2+^, Zn ^2+^ and Cd^2+^ to show different colors in plants flower (Kondo et al., 1998). Metalloanthocyanins theory holds that anthocyanins, flavones, and metal ions fix at 6: 6: 2 in blue flowers (Takeda, 2006). With biological development, more and more flower color can be explained by genes or metabolite levels. Nowadays, scientists believe that flower color is predominantly controlled by the production of flavonoids, carotenoids, and alkaloid-related compounds, such as betalains (Holton, 1995). For example, researchers have found that yellow flower petals often contain yellowish xanthophylls, β-carotenoids, and chrysanthemum. All of them belong to the class of carotenoids (Nielsen et al., 2003; Kishimoto et al., 2004). Some species such as roses, and carnations are lacking blue because of the absence of Flavonoid3’,5’-hydroxylase, a key enzyme catalyzing the hydroxylation reaction between dihydrokaempferol and dihydromyricetin (Holton and Cornish, 1995; Kondo et al., 1998).

*M. candidum* belongs to the Melastomataceae family which is mainly centered in Southeast Asia and extends to India, South China, and Northern Australia (Liu et al., 2014). Although many species of Melastomataceae have a relatively high degree of overlap in geographic distributions and flower periods, some members still face reproductive isolation problems. Additionally, many members of the Melastomataceae are dull colors, which limits their use in garden industries. Here, we took *M. candidum* as a research object and conducted lncRNA-seq, sRNA-seq, mRNA-seq, and a widely targeted metabolome for three development stages of the flowers. Based on differentially expressed RNA analyses, we developed three functional acting models, including antisense, *cis*, and trans - acting models. Two networks of antisense and *cis* models were constructed in flavonoid, anthocyanin, carotenoid, and alkaloid-related pathways. According to the ceRNA theory, a lncRNA-mediated regulatory network was also built in the aforementioned pathways. A correlation among all kinds of RNAs and metabolites was conducted to illustrate the relationship among them. This research aims to unveil the role of lncRNA in flower development in *M*. *candidum*.

## 2 Materials and methods

### 2.1 RNA extraction, cDNA library construction, and sequencing

Three developmental stages of the flower of *M. candidum* including closed buds with white petals (McI), closed buds with pink petals (McII), and opened buds with pink petals (McIII) were collected and immediately frozen in liquid nitrogen. Three replicates of each stage sample were taken from three seedlings. Total RNA was extracted by using an OminiPlant RNA Kit (DNase I) (CW2598, CWBIO, Taizhou, China) according to the manufacturer’s protocol. An Agilent 2100 Bioanalyzer was utilized to assess the quality of the RNA (Agilent Technologies, Palo Alto, CA, USA). To generate a cDNA library, we fragmented the mRNA using a fragmentation buffer and reverse-transcribed the resulting small fragments into cDNA using random primers. The second strand cDNA was synthesized by employing DNA polymerase I, RNaseH, dNTP, and buffer. After purification with the poly(A) and PCR extraction kit (Qiagen), the synthesized products were further purified using the QiaQuick PCR extraction kit (Qiagen, Venlo, The Netherlands) and then ligated to Illumina sequencing adapters. Afterward, the second-strand cDNA was digestedusing the enzyme UNG (Uracil-N-Glycosylase), and the resulting products were separated by size on an agarose gel before being amplified by PCR. The PCR products were then sequenced on Illumina HiSeq TM 4000 platforms.

### 2.2 Filter of the raw data and alignment against the genome

After obtaining sequence data, raw reads consisting of adapters or low-quality bases were filtered by the fastp software (version 0.18.0) (Chen et al., 2018b) with steps: 1) removing reads containing adapters; 2) removing reads containing more than 10% of unknown nucleotides (N); 3) removing low quality reads containing more than 50% of low quality (Q-value ≤ 20) bases. After filtering, the clean short reads were aligned to the ribosome RNA (rRNA) database using Bowtie2 (version 2.2.8) (Langmead and Salzberg, 2012) to eliminate the rRNA-mapped reads. After building an index of the *M. candidum* genome, the paired-end clean reads were mapped to the reference genome by using HISAT2 (version 2.1.0) (Kim et al., 2015) with a default parameter “-rna-strandedness RF”.

### 2.3 Transcripts reconstruction and annotation

The reconstruction of transcripts was carried out with the software Stringtie (version 1.3.4) (Pertea et al., 2015). To identify the novel transcripts among the reconstructed transcripts, all the reconstructed transcripts were aligned to the reference genome and were then categorized into 12 categories using the Cuffcompare program (Trapnell et al., 2010). A novel transcript was defined if it had one of the following class codes - u, i, j, x, c, e, or o. To further identify new genes, we used the following parameters: length of transcript > 200 bp, number of exons > 1 (Lu et al., 2019). All the novel transcripts were then aligned to the Nr, KEGG, and GO databases to obtain protein functional annotations.

### 2.4 lncRNA prediction and classification

Software including Coding-Non-Coding Index (CNCI, version2) (Sun et al., 2013) and Coding Potential Calculator 2 (CPC2, version 0.9-r2) (Kong et al., 2007) (http://cpc.cbi.pku.edu.cn/) were used to assess the protein-coding potential of novel transcripts. Only transcripts which meet the protein-coding-score criteria (CNCI sequence-score < 0 and CPC2 coding probability < 0.5) were considered as lncRNAs. Based on the location relative to protein-encoding genes (PCG), lncRNAs are categorized into five classes: intergenic lncRNAs (located between two PCGs), bidirectional lncRNAs (situated on the opposite strand but within 1 kb of the promoter on the sense strand), intronic lncRNAs (located within an intron of a PCG on the sense strand), antisense lncRNAs (transcribed from the opposite strand of a PCG), and sense lncRNA (spanning multiple introns or exons within a PCG) (Ahmad et al., 2021).

### 2.5 Quantification of transcripts and differentially expressed analysis

The abundance of the transcript was quantified using StringTie (Pertea et al., 2015). An FPKM (fragment per kilobase of transcript per million mapped reads) value was calculated for each transcription region to mitigate the effect of varying transcript lengths and sequencing data amounts on expression. DEGs of coding RNAs and lncRNAs were analyzed separately by using DESeq2 software (Li and Dewey, 2011) between two versus groups by the following steps: 1) normalization of read counts; 2) calculation of p-value; 3) correction of value to get a false discovery rate (FDR) value. Differentially expressed lncRNAs were screened with the threshold: Fold change ≥ 2, FDR ≤ 0.05.

### 2.6 Differential analyses of miRNAs, mRNAs, and metabolites

For miRNAs, expression level was calculated and normalized by transcripts per million (TPM) methods. EdgeR (Robinson et al., 2010) were used to conducted differential analysis. DEmiRNAs were identified with the absolute threshold log_2_ (fold change) ≥ 0.585, *p*-value ≤ 0.05. For mRNAs, HISAT2 (Kim et al., 2015) were used to align to the genome of *M*. *candidum* (http://evolution.sysu.edu.cn/Sequences.html). The FPKM value was used to quantify expression abundance and variations. DEmRNAs were identified by DESeq2 (Love et al., 2014) package with the absolute threshold log_2_ (fold change) ≥ 1 and *p*-value ≤ 0.05. Metabolites were analyzed by using an LC-ESI-MS/MS system (UPLC, Shim-pack UFLCSHIMADZU CBM30A, http://www.shimadzu.com.cn/; and MS/MS (Applied Biosystems 6500 QTRAP, http://www.appliedbiosystems.com.cn/) (Chen et al., 2013) and variable importance in projection (VIP) score of (O)PLS model was applied to rank the metabolites and distinguish two versus groups. DSmets were identified with the absolute threshold log_2_ (fold change) ≥ 0.585, vip ≤ 1.

### 2.7 LncRNA-mRNA association analysis

In order to identify three direct-acting models of lncRNAs and mRNAs including antisense-regulation, *cis*-regulation, and trans-regulation, the software RNAplex (version 0.2) (Shen et al., 2014) (http://www.tbi.univie.ac.at/RNA/RNAplex.1.html) was used to predict the complementary correlation and detect the interaction between antisense lncRNAs and mRNAs. Antisense lncRNAs can form complementary base pairing with mRNAs. The ViennaRNA package (Lorenz et al., 2011) in R software was used to predict the best base pairing based on the calculation of minimum free energy through thermodynamics structure. *Cis* lncRNAs could regulate neighboring genes on the same allele. LncRNAs with the unknown region were annotated again to identify *cis*-regulators. Trans lncRNAs could regulate co-expressed genes far from them. The correlation of expression between lncRNAs and protein-coding genes was used to identify a trans-regulation relationship with a Pearson correlation of more than 0.999.

### 2.8 Validation of lncRNA-seq result by qRT-PCR

In order to verify the lncRNA-seq result, we chose 12 lncRNAs to perform qRT-PCR. The primers of lncRNAs used in qRT-PCR were listed in **Table S1**. 0.5 µg total RNA of three stages flowers was reverse-transcribed into first-strand cDNA using the PrimeScript RT reagent Kit gDNA Eraser (Takara, Dalian, China). Then, the SYBR @Premix Ex Taq TMII (Takara, Dalian, China) was used according to the manufacturer’s instruction for qRT-PCR of lncRNAs on the Illumina Eco real-time PCR system (Illumina, USA). The α -tubulin gene of *M*. *candidum* was used as the internal reference gene. Ct values were then calculated by the 2^−ΔΔCt^ algorithm. Primers sequences, length of PCR products and PCR amplification efficiency for each pair of primers are listed in **Table S1**. Relative expression levels of selected lncRNAs at different stages of flowers were analyzed using One-way ANOVA with multiple comparison by GraphPad Prism 9. The graphs were visualized by GraphPad Prism 9 and Adobe Illustrator 2020.

### 2.9 Construction and validation of lncRNA-mediated ceRNA network

The ceRNA network was constructed based on the following rules: 1) negative correlation between miRNA and lncRNAs, as well as miRNA and mRNAs with calculation of Spearman correlation coefficient (SCC); 2) positive correlation between lncRNAs and mRNAs with calculation of Spearman correlation coefficient (SCC); 3) enrich degree of lncRNA binding to miRNAs and miRNAs binding to mRNAs. Relationships among ceRNA members and metabolites were visualized by the ggalluvial package in R3.6.1 software.

To verify the lncRNA-miRNA-mRNA regulatory chains, the transient coexpression experiments were performed in leaves of *Nicotiana benthamiana* according to Lu et al. (Lu et al., 2019). Precursors of miRNA (pre-miRNA), lncRNAs, and mRNAs were cloned from mixture of cDNA of three-stages flowers. For miRNAs that could not obtain pre-miRNAs in the cDNA, we performed overlap PCR to ligate these mature miRNAs into the vector. Pre-miRNAs and lncRNAs were ligated into pCAMBIA2300 at *Sma*I and *Xba*I sites. mRNAs were ligated into pCAMBIA1300 at *Xba*I and *Kpn*I sites. The vector harboring pre-miRNAs, lncRNAs, and mRNAs were then transformed into *Agrobacterium tumefaciens* GV3101 strain. Equal amounts of agrobacterial cell cultures containing miRNAs, lncRNAs, and mRNAs were mixed respectively. Then, the mixture was infiltrated into leaves of *N*. *benthamiana*. After being incubated in dark for two days, the infiltrated tobacco leaves were observed under laser scanning confocal microscopy (LSCM) with EGFP = 780, and collected for total RNA extractions (CW2598, CWBIO, Taizhou, China) and qRT-PCR (Takara, Dalian, China). Tobacco L23 was used as an internal reference (Liu et al., 2012). The primers were listed in **Table S1**.

## 3 Results

### 3.1 Statistics information of sequenced lncRNAs

**Figure 1:**
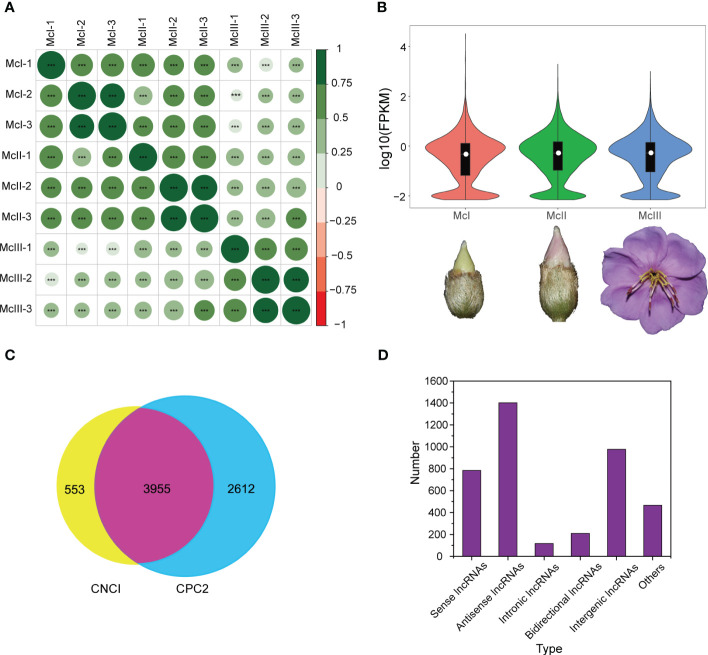
Basic statistics information of lncRNAs sequencing. **(A)** Pearson correlation analysis among different samples. Green and big size mean high correlation coefficient. “***” means a P value less than 0.001. **(B)** Violin graph reflecting expression distribution of samples in the different developmental stages. White dots in the middle of the violins represent the median value of the expression level. **(C)** Venn graph of two lncRNA prediction software. **(D)** Detected numbers of different lncRNAs categories.

Prior to conducting differentially expressed analyses, we performed basic statistical analyses on the sequencing data, such as examining correlations between samples, evaluating expression distributions, and identifying types of lncRNAs. Sample correlation was determined based on the expression level of lncRNAs (**Figure 1A**). Correlations among samples of each stage had a relatively high coefficient, indicating these samples meet our analysis requirements. A relatively high average expression level was observed at the late stage (**Figure 1B**). In order to increase the reliability of the results, we used CNCI and CPC2 to identify lncRNAs in sequenced samples. A total of 4,508 lncRNAs were identified by CNCI and a total of 6,567 lncRNAs were identified by CPC2. To ensure greater accuracy, we took the intersection of the results from both software and obtained 3,955 lncRNAs (**Figure 1C**). Five types of lncRNA were identified, including sense lncRNAs, antisense lncRNAs, intronic lncRNAs, bidirectional lncRNAs, and intergenic lncRNAs. It should be noted that some lncRNAs in the graph cannot be assigned a precise category. Among the identified lncRNA, the proportion of antisense lncRNAs was the highest, while that of intronic lncRNAs was the lowest (**Figure 1D**). Overall, the lncRNAs were suitable for further analysis.

### 3.2 Differentially expressed analyses of lncRNAs

**Figure 2:**
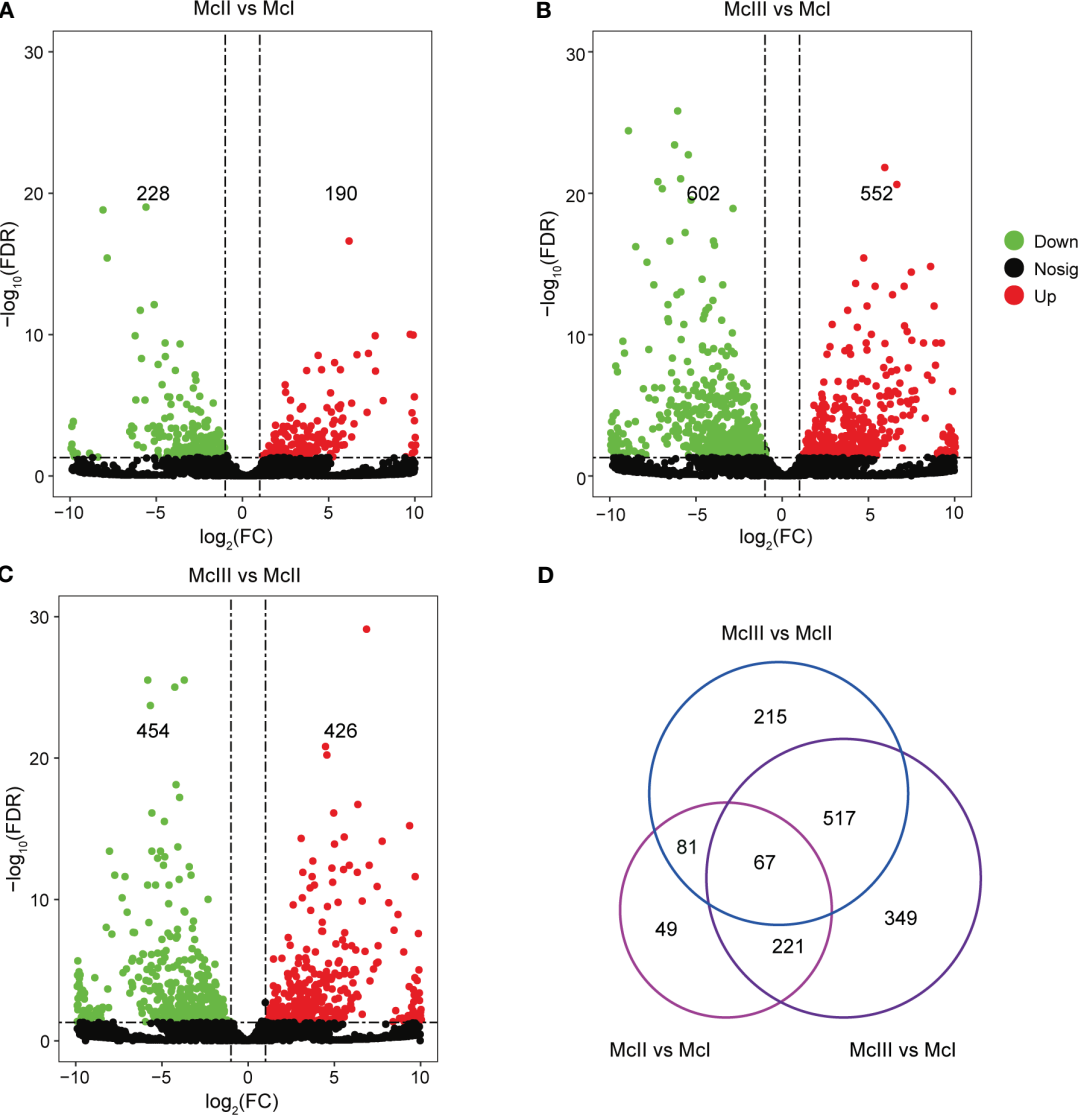
Differentially expressed lncRNAs (DElncRNAs) in different comparing groups. **(A)** DElncRNAs in stageII (McII) versus stageI (McI) group. **(B)** DElncRNAs in stageIII (McIII) versus stageI (McI) group. **(C)** DElncRNAs in stageIII (McIII) versus stageII (McII) group. Red dots mean up-regulated lncRNAs, and green dots mean down-regulated lncRNAs. **(D)** Venn graphs of different versus groups.

For all lncRNAs identified, we performed differential expression analyses among different stages (**Figure 2**). There were 190 up-regulated and 228 down-regulated lncRNAs in the McII vs McI group (**Figure 2A**; **Table S2**). In the comparison between the McIII and McI group, we identified 552 up-regulated and 602 down-regulated lncRNAs (**Figure 2B**; **Table S3**). 426 lncRNAs were up-regulated and 454 lncRNAs were down-regulated in the McIII vs McII group (**Figure 2C**; **Table S4**). Differential expression analyses results indicated huge differences of lncRNA expression levels between McIII and the other two stages. Then, we gave a statistics among the three comparison groups using Venn method which yieled 67 common lncRNAs (**Figure 2D**). As lncRNAs play a variety of roles during the different stages of development, we used the union of three comparison groups to conduct further analysis and identify the roles of lncRNAs during the different stages of flower development.

### 3.3 Antisense, *cis*, and trans-acting model of lncRNAs and functional annotation of their targeted mRNAs

**Figure 3:**
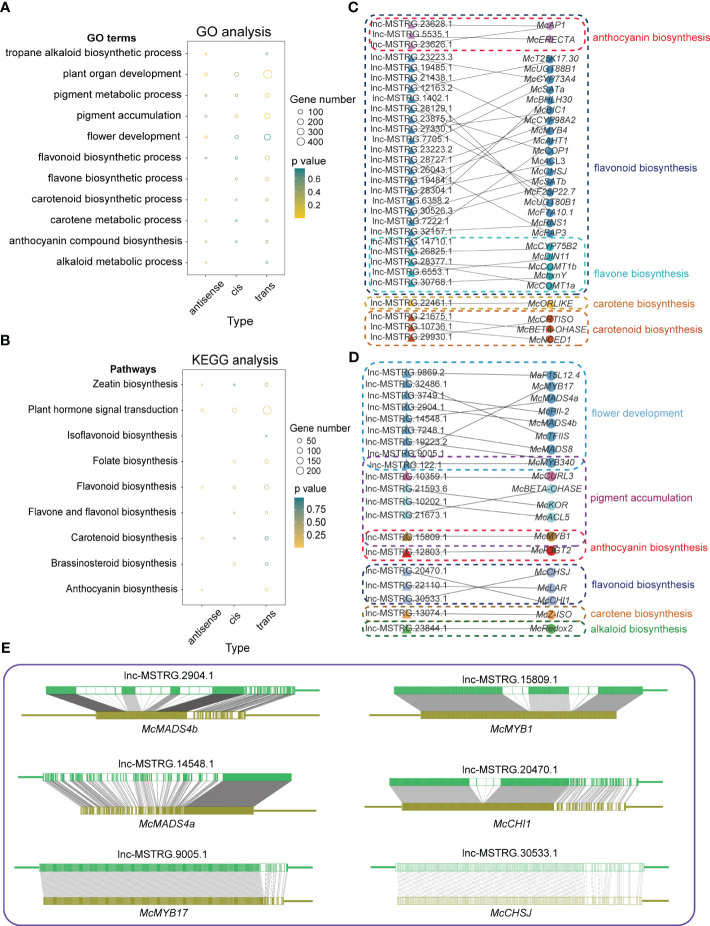
Different interacting models of lncRNAs with mRNAs. **(A)** GO analysis of three interacting models. Only biological process terms are shown in this graph. **(B)** KEGG analysis of three interacting models. In both graphs, A,B the size of the circles means enriched gene numbers in different terms or pathways, and orange means significantly enriched. **(C)** A network of *cis* model between lncRNAs and mRNAs in flower development and flower color formation related pathways. **(D)** A network of antisense model between lncRNAs and mRNAs in flower development and flower color formation related pathways. **(E)** The complementary relationship between lncRNAs and mRNAs in the antisense model.

It has been reported that lncRNAs interact with mRNAs in three distinct ways including *cis*, trans, and antisense interactions (Nie et al., 2012). *Cis* model means that lncRNAs could induce reconstruction and histone modification to influence the combination of transcript factors with promoter and enhancer or combine with transcription elements directly to affect the expression of the protein-encoding genes. In the antisense model, it is assumed that lncRNAs could directly bind to mRNAs to influence alternative splicing of mRNAs or to decay mRNAs into siRNAs, which is closely related to the stability of mRNAs. The trans model is often able to be judged by the opposite expression trends between lncRNAs and their mRNA counterparts. LncRNAs could sometimes bind to proteins translated by mRNAs, altering their activity, structure, or position to influence their corresponding mRNAs. Here we also confirmed three functional models for differentially expressed lncRNAs and then GO and KEGG analyses were performed for their corresponding mRNAs (**Figures 3A, B**; **Tables S5-S10**). In **Figures 3A, B**, some GO terms associated with flower development and color formation were chosen for visualization, including alkaloids, pigments, flavonoids, hormones, and flower development-related terms or path. As a result of the three acting models, the trans model enriched more genes than the other two functional models. However, we could not find the antisense models of these DElncRNAs in the flavone biosynthetic process of the result of GO analysis as well as the isoflavonoid biosynthetic process, the folate biosynthesis, and the brassinosteroid biosynthetic process of the results of KEGG analysis (**Figures 3A, B**). Similarly, there was also lack of *cis* models of these DElncRNAs in the Tropane Alkaloid biosynthetic process and the Alkaloid metabolic process of the results of GO analysis, and the isoflavonoid biosynthesis and Anthocyanin biosynthesis of the results of KEGG analysis (**Figures 3A, B**).

To further investigate the potential roles of lncRNAs in regulating gene expression by antisense and *cis* models, we built lncRNAs-target genes networks (**Figures 3C, D**). In the *cis* model, we identified 26 lncRNAs that function on genes involved in flavonoid biosynthesis. Of these, three lncRNAs, lnc-MSTRG.23628.1, lnc-MSTRG.5535.1, and lnc-MSTRG.23626.1, were related to anthocyanin biosynthesis, while five lncRNAs, lnc-MSTRG.14710.1, lnc-MSTRG.26825.1, lnc-MSTRG.28377.1, lnc-MSTRG.6553.1, and lnc-MSTRG.30768.1 were related to flavone biosynthesis. In addition to flavonoid biosynthesis, we found one lncRNA in carotene biosynthesis and three lncRNAs in carotenoid biosynthesis (**Figure 3C**). In contrast to the *cis* model, we observed fewer lncRNAs involved in flavonoid biosynthesis in the antisense model, with only three lncRNAs, lnc-MSTRG.15809.1, lnc-MSTRG.12803.1, and lnc-MSTRG.30533.1, were detected. No lncRNAs involved in flavone biosynthesis process were identified (**Figure 3D**). Overall, our findings suggest that there are more *cis* models than antisense models involved in regulating flower development and color formation in *M*. *candidum*. The antisense model involves lncRNAs forming complementary relationships with genes. To investigate this further, we examined several genes, *McMADS4a*, 4b, *McMYB1*,*17*, *McCHSJ*, and *McCHI1*, along with their corresponding lncRNAs to assess their complementary relationship. Our results showed a high degree of complementarity between the lncRNAs and their target genes (**Figure 3E**).

### 3.4 Validation and expression model of lncRNAs in three development stages of *M. candidum*

**Figure 4:**
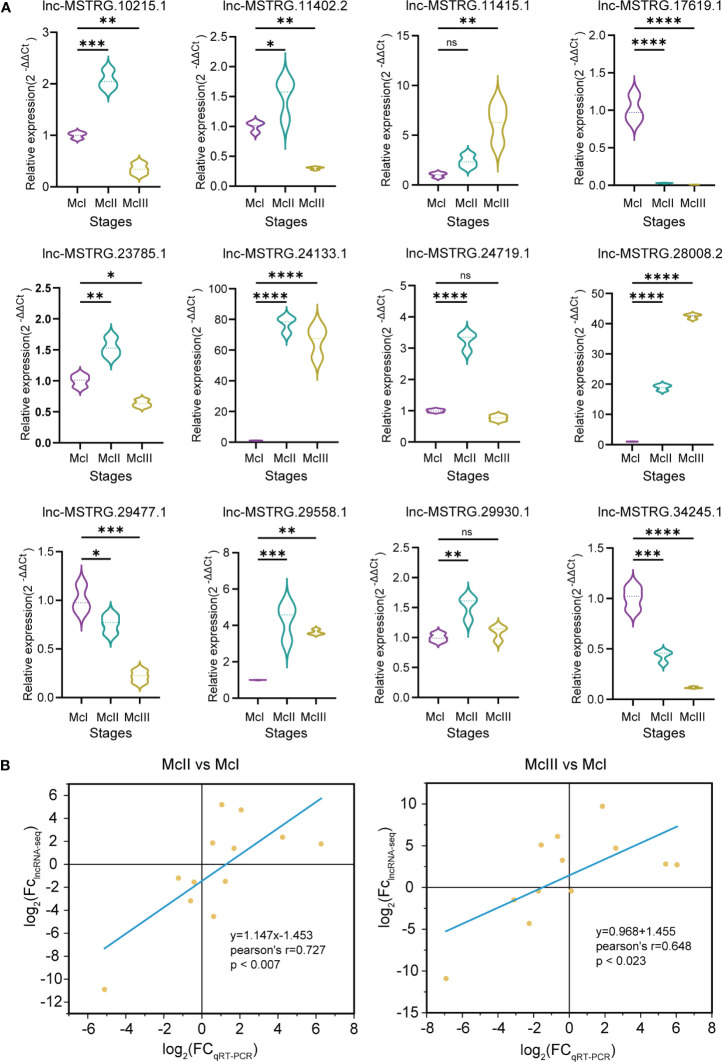
Validation of lncRAN-seq results. **(A)** qRT-PCR results of 12 selected lncRNAs. Three colors represent three stages. Dot lines within the violin graphs mean median values. "ns" means p >0.05; "*" means p ≤ 0.05; "**" means p ≤ 0.01; “***” means p ≤ 0.001; “****” means p ≤ 0.0001. **(B)** Fitting result bewtween qRT-PCR and lncRNA-seq. In both qRT-PCR and miRNA-seq results, fold change (FC) was nomorlized by log_2_ algorithm.

In order to confirm the accuracy of our lncRNA-seq data, we performed qRT-PCR assays on 12 selected lncRNAs. The expression patterns of most of these lncRNAs were found to be consistent with the results obtained from the sequencing analysis. Specifically, lnc-MSTRG.17619.1, lnc-MSTRG.29477.1, and lnc-MSTRG.34245.1 were highly expressed during the McI stage, while lnc-MSTRG.10215.1, lnc-MSTRG.11402.2, lnc-MSTRG.223785.1, lnc-MSTRG.24719.1, lnc-MSTRG.29558.1, and lnc-MSTRG.29930.1 were highly expressed during the McII stage **Figure 4A**. Additionally, lnc-MSTRG.28008.2 and lnc-MSTRG.11415.1 were highly expressed during the McIII stage, while lnc-MSTRG.24133.1 was highly expressed in both the McII and McIII stages **Figure 4A**. We found that the results obtained from the lncRNA-seq analysis and the qRT-PCR experiments were positively correlated, with a slope of 1.147 and an R value of 0.727 for the McII vs McI group, and a slope of 0.968 and an R value of 0.645 for the McIII vs McII group (as shown in **Figure 4B**). Based on these findings, we concluded that the lncRNA-seq analysis was valid and accurate.

**Figure 5:**
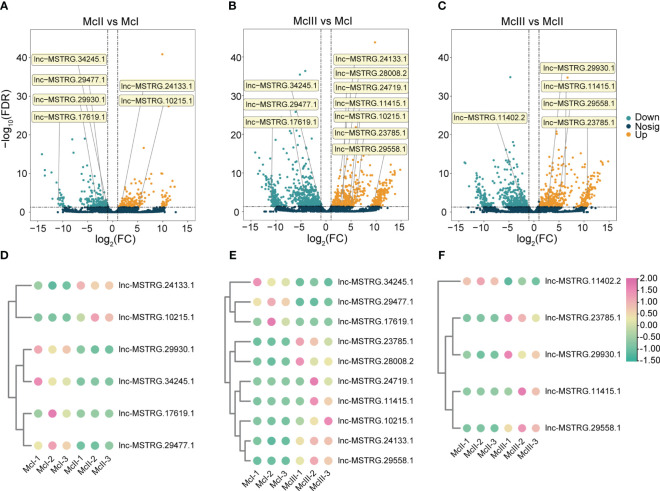
Distribution and expression heatmaps of selected lncRNAs. **(A)** Selected lncRNAs in McII vs McI group. **(B)** Selected lncRNAs in McIII vs McI group. **(C)** Selected lncRNAs in McIII vs McII group. In **(A–C)**, orange means up-regulated lncRNAs, and green means down-regulated lncRNAs. **(D)** Heatmap of selected lncRNAs in McII vs McI group. **(E)** Heatmap of selected lncRNAs in McIII vs McI group. **(F)** Heatmap of selected lncRNAs in McIII vs McII group. In graphs **(D–F)**, pink dots mean high expression level, and the light green dot means low expression level.

We investigated the expression pattern of these lncRNAs during different stages of the flower development process. In the McII vs McI group, a total of six DElncRNAs were confirmed, of which four were down-regulated and two were up-regulated (**Figures 5A, D**). Ten DElncRNAs were differentially expressed between the McIII and McI groups, of which three were downregulated and seven were upregulated (**Figures 5B, E**). Five DElncRNAs related to flower color formation were found between McIII and McII, including one that was down-regulated and four that were up-regulated (**Figures 5C, F**). During the transition from the second to the third stage of flower development, lnc-MSTRG.10215.1 and lnc-MSTRG.24133.1 were up-regulated and exhibited an upward trend in expression, indicating their corresponding competing endonous mRNAs were key regulator in regulating this period of development. Conversely, lnc-MSTRG.34245.1, lnc-MSTRG.29477.1, and lnc-MSTRG.17619.1 showed a decline in expression from the second stage onwards (**Figures 5A, B**), indicating their corresponding competing endonous mRNAs were less involved in this process. It was observed that lnc-MSTRG.11415.1 and lnc-MSTRG.29558.1 began to be highly expressed in the third period (McIII), suggesting that these two lncRNAs will play a critical role in flower development after color formation (**Figures 5B, C, E, F**). There is only a low expression of lnc-MSTRG.11402.2 in the second stage, showing that its target gene plays a negative role in flower coloration process.

### 3.5 LncRNAs-mediated ceRNA regulatory network build and verification

**Figure 6:**
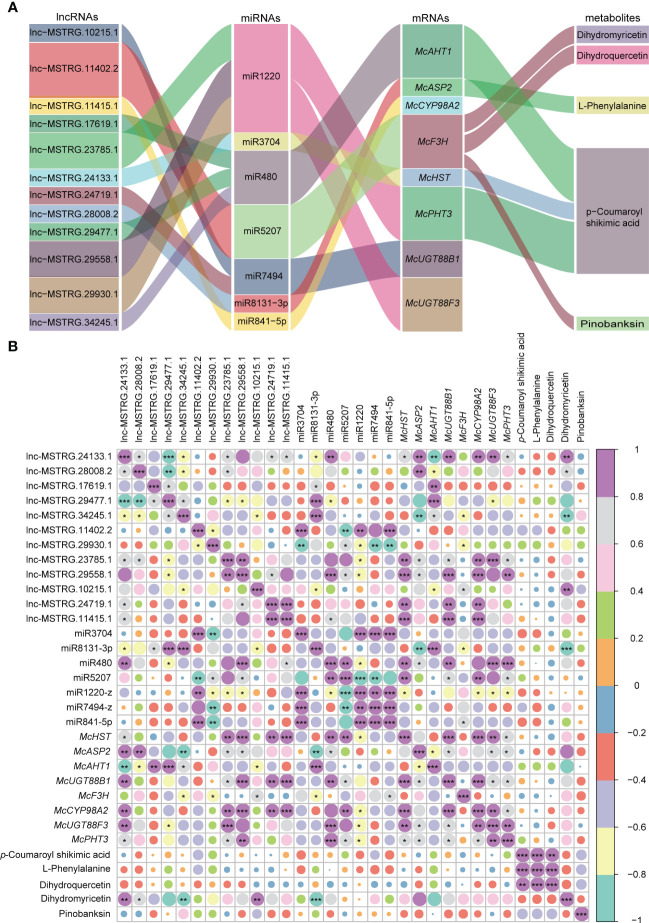
Sankey and correlation relationship map of selected lncRNAs, miRNAs, mRNAs, and metabolites. **(A)** Corresponding relationship among lncRNAs, miRNAs, mRNAs, and metabolites. **(B)** Correlation relationship among lncRNAs, miRNAs, mRNAs, and metabolites. Purple means a high correlation coefficient, and green means a low correlation coefficient. “***” means *P*-value is significant at 0.001 level, “**” means the *P*-value is significant at 0.01 level, and “*” means *P*-value is significant at 0.05 level.

To further clarify the regulatory mechanism of the aforementioned lncRNAs, we first examined the supplementary relation between lncRNAs and miRNAs, miRNAs and target genes (**Figure S1**). Subsequently, we constructed a ceRNA regulatory network, which included lncRNAs, miRNAs, mRNAs, and metabolites for flower formation-related pathways (**Figure 6A**). As shown in the graph, lnc-MSTRG.10215.1 and lnc-MSTRG.11402.2 were found to regulate *McF3H* through interacting with miR5207, ultimately influencing dihydromyricetin (DHM), dihydroquercetin (DHQ), and pinobanksin (PBA). Dihydroquercetin (DHQ), also known as 3, 5, 7, 3, 4 - pentahydroxy flavanone or taxifolin, is a bioactive flavonoid that is considered one of the rarest and most effective natural antioxidants (Yu et al., 2021). Both dihydroquercetin and dihydromyricetin are colorless dihydroflavones that can be reduced to leucoanthocyanidins under the action of the dihydroflavonol-4-reductase (DFR) enzyme. Subsequently, under the catalysis of downstream enzymes, the leucoanthocyanidins undergo a process of the transformation into orange pelargonidin, reddish-purple cyanidin, and violet-blue delphinidin (Tanaka and Brugliera, 2013; Liu et al., 2019). Pinobanksin, one of the most common phenolic constituents of pine heartwood, was first isolated from *Pinus Banksiana* (Erdtman, 1944). When combined with zinc ion, this compound is specific to 3-hydroxyflavanones to produce much deeper colors during the reducing process. Pinobanksin usually produces orange-red when reduced with magnesium or zinc and hydrochloric acid (Lindstedt, 1950). *P*-coumaroyl Shikimic acid is a by-product of the anthocyanin pathway, derived from 4-coumaroyl-CoA by the formation of an ester bond with shikimic acid catalyzed by Shikimate O-hydroxycinnamoyl transferase (HCT) (Paliyath et al., 2009; Li et al., 2021). In the presence of the enzymes C4H1 and C3H3, this compound can be catalyzed again to transform into caffeoyl shikimic acid (Chen et al., 2011). Both *p*-coumaroyl Shikimic acid and caffeoyl shikimic acid are key compounds in Phenylpropanoid biosynthesis during lignin formation. The Sankey graph showed that miR3704, miR480, and miR1220 had cleavage effects on *McHST*, *McAHT1*, and *McPHT3*, respectively. Additionally, we discovered that lnc-MSTRG.24133.1 could adsorb miR3704 to maintain a balance between miRNA and target gene. Three lncRNAs, lnc-MSTRG.17619.1, lnc-MSTRG.29477.5, and lnc-MSTRG.34245.1, were found to bind to miR480 while another set of three lncRNAs, lnc-MSTRG.23785.1, lnc-MSTRG.29558.1, and lnc-MSTRG.29930.1 were found to bind to miR1220. These results indicate that the three lncRNAs that ceRNA regulatory mechanism play an important role in the formation of *p*-coumaroyl Shikimic acid, which ultimately determine flower color. Three specific genes, namely *McUGT88B1*, *McUGT88F3*, and *McCYP98A2*, were found to have no associated with metabolites. This may indicate that the three genes related to ceRNAs do not significant affect metabolism. It is also possible that annotation of the metabolite database is incomplete.

We conducted a correlation analysis to determine the relationship between ceRNA members and metabolites in specific pathways (**Figure 6B**). Each ceRNA regulatory routine showed a negative correlation between miRNAs and both lncRNAs and mRNAs, whereas a positive correlation was observed between lncRNAs and mRNAs, indicating the presence of a true competitive mechanism between lncRNAs and mRNAs. Lnc-MSTRG.28008.2 and *McASP2* were negatively correlated with L-phenylalanine, however, miR8131-3p was positively correlated with L-phenylalanine, indicating that this ceRNA regulatory chain is responsible for L-phenylalanine synthesis (**Figure 6B**). It is known that both the flavonoid and anthocyanin pathways originate from the amino acid L-phenylalanine, which is deaminated by the action of phenylalanine ammonia-lyase (PAL) to produce trans-cinnamic acid and ammonia (Fasoula et al., 1995). Lnc-MSTRG.29477.1, lnc-MSTRG.29558.1, *McAHT1*, and *McPHT3* were positively correlated with *p*-coumaroyl Shikimic acid, whereas, miR480 and miR1220 were negatively correlated with *p*-coumaroyl Shikimic acid, indicating that these two ceRNAs regulatory chains were key regulator in the synthesis of *p*-coumaroyl Shikimic acid. However, the rest of ceRNA regulatory networks did not display obvious putative correlations, which could be attibuted to more complex regulatory mechanisms like multiple ceRNA chains, histone modification, RNA methylation modification process, *etc*. Further studies are needed to gain a better understanding of these complex regulatory mechanisms.

**Figure 7:**
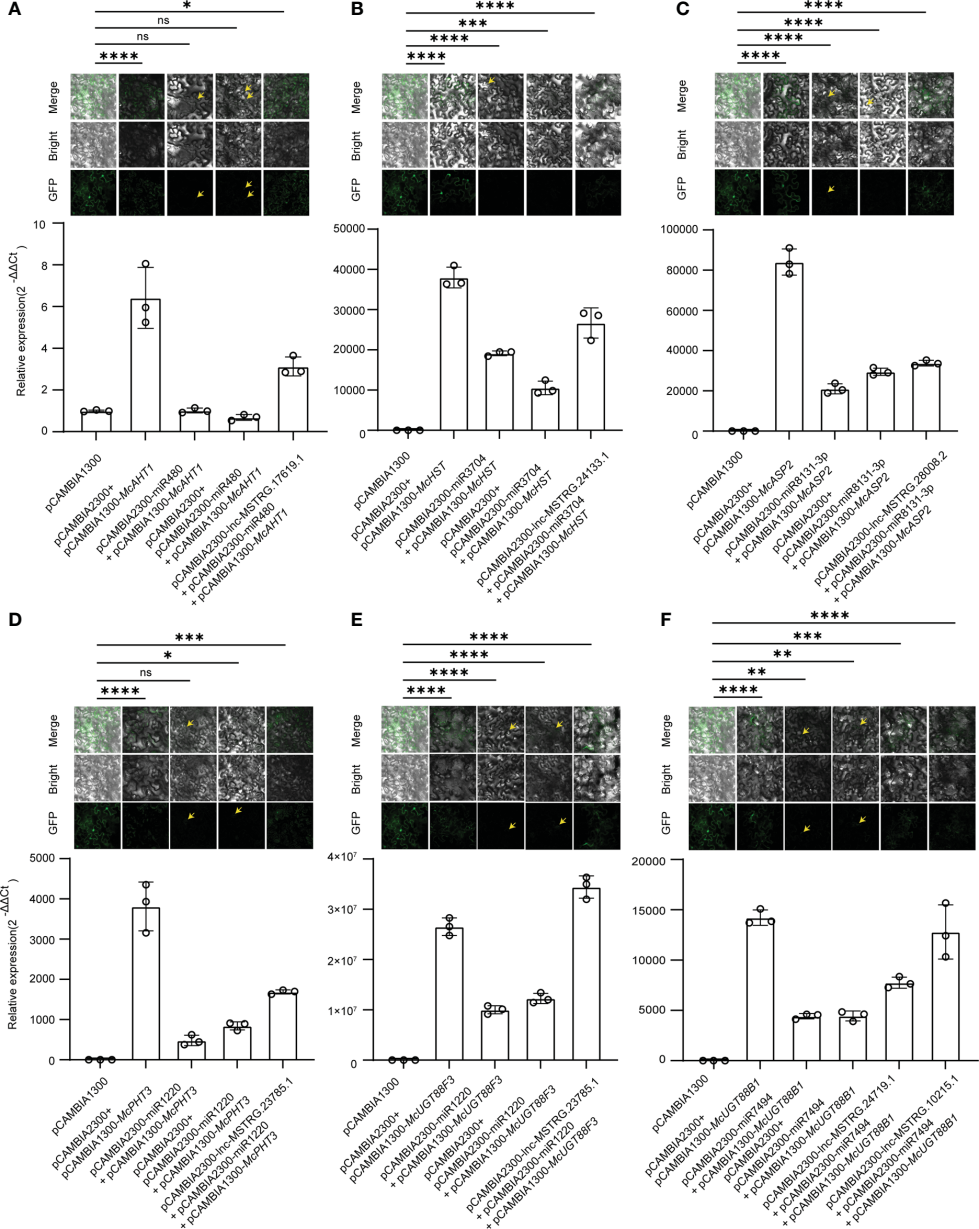
Validation of lncRNA-Mediated ceRNA regulatory network. **(A)** Validation of lnc-MSTRG.17619.1-miR480-McAHT1 regulatory chain. **(B)** Validation of lnc-MSTRG.24133.1-miR3704-McHST regulatory chain. **(C)** Validation of lnc-MSTRG.28008.2-miR8131-3p-McASP2 regulatory chain. **(D)** Validation of lnc-MSTRG.23785.1-miR1220-McPHT3 regulatory chain. **(E)** Validation of lnc-MSTRG.23785.1-miR1220-McUGT88F3 regulatory chain. **(F)** Validation of lnc-MSTRG.24719.1-miR7494-McUGT88B1 and lnc-MSTRG.10215.1-miR7494-McUGT88B1 regulatory chains. The pictures above column are GFP fluorescence graphs obtained using a laser scanning confocal microscope (LSCM). “*” means p ≤ 0.05; “**” means p ≤ 0.01; “***” means p ≤ 0.001; “****” means p ≤ 0.0001; “ns” means not significant. Yellow arrows point to weak fluorescence in the images.

To further validate the identified lncRNA-mediated ceRNA regulatory network, we employed pCAMBIA1300 harboring target genes, and pCAMBIA2300 harboring lncRNAs and miRNAs to performed tobacco injection and qRT-PCR experiment. Ordinarily, miRNAs have cleavage effects on the target genes, when lncRNAs bind with miRNAs, the cleavage effects will be weakened. We selected 7 lncRNA-mediated ceRNA regulatory routines which included 6 genes, 5 miRNAs, and 6 lncRNAs to validate the ceRNA regulatory mechanism in tobacco (**Figure 7**). In accordance with our anticipation, when only injecting the miRNAs and target genes into leaves of tobacco, the fluorescence signal became weak (**Figure 7**), and the expression level of target genes begun to decrease. When we added the lncRNAs in corresponding ceRNA chains, the fluorescence signal was regained, and the expression level of the target gene also began to increase (**Figure 7**). These results provided evidence for the involvement of lncRNA-mediated ceRNA regulation in *M*. *candidum*.

### 3.6 Functional model of lncRNA-mediated ceRNA during flower development of *M. candidum*

**Figure 8:**
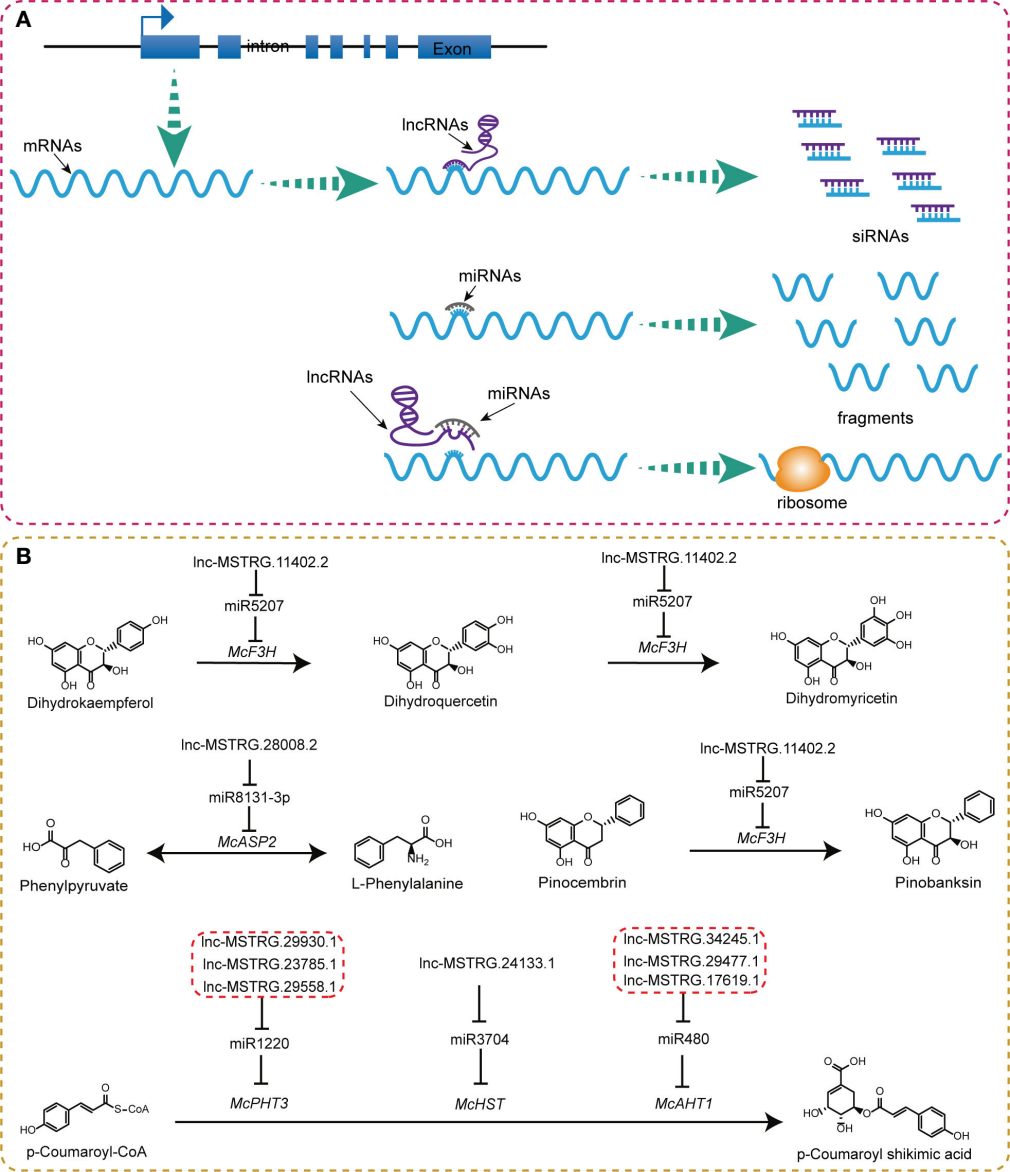
LncRNAs-mediated ceRNAs regulatory mechanism in flavonoids pathway. **(A)** Functional model of lncRNA . **(B)** Location of lncRNA-mediated ceRNA regulatory chains in corresponding compound biosynthesis process of flavonoids pathway.

Based on the aforementioned results, we summarized some key lncRNA-mediated ceRNA regulatory relationship during flower development (**Figure 8**; **Figure S2**). LncRNAs could bind with a post-transcriptional product of genes directly to degrade them into siRNAs (small interference RNAs). Another common path of lncRNAs is to compete with mRNAs to bind to miRNAs, thereby easing the cleavage effect of miRNA and facilitating more efficient translation of mRNAs (**Figure 8A**). We observed some ceRNA regulatory mechanisms operating in the flavonoid pathway. Specifically, lnc-MSTRG.11402.2-miR5207-*McF3H* formed a regulatory chain that acts as a catalyst for the transformation from pinocembrin to pinobanksin (**Figure 8B**). Lnc-MSTRG.28008.2-miR8131-3p-*McASP2* catalyzes the conversion of phenylpyruvate into L-phenylalanine. Lnc-MSTRG.29930.1, lnc-MSTRG.23785.1, and lnc-MSTRG.29558.1 could bind with miR1220 to regulate *McPHT3* genes; lnc-MSTRG.24133.1 could affect *McHST* by interacting with miR3704; Lnc-MSTRG.34245.1, lnc-MSTRG.29477.1, and, lnc-MSTRG.17619.1 completed with miR480 to bind to *McAHT1*. All these three combinations together influenced the metabolic process from *p*-Coumaroyl-CoA to *p*-Coumaroyl shikimic acid (**Figure 8B**). Out of all the observed lncRNA-miRNA-mRNA-metabolites regulatory chains, lnc-MSTRG.29930.1, *McPHT3*, and *p*-Coumaroyl shikimic acid were lowly expressed in the second stage, whereas miR1220 was highly expressed in the second stage, indicating the presence of a ceRNA regulatory mechanism. It was found that lnc-MSTRG.24133.1, *McHST*, and *p*-Coumaroyl shikimic acid were highly expressed in the third stage, but miR3704 was lowly expressed, suggesting that this ceRNA regulatory chain also plays an important role in the biosynthesis of *p*-Coumaroyl shikimic acid (**Figure S4**). We also found some irregular ceRNA regulatory chains, for example, lnc-MSTRG.11402.2-miR5207-*McF3H*, which was thought to be involved in the synthesis of dihydroquercetin, dihydromyricetin, and pinobanksin. However, we did not observe any corresponding expression trends for these metabolites; lnc-MSTRG.17619.1, lnc-MSTRG.29477.1, lnc-MSTRG.34245.1-miR480-*McAHT1*-*p*-Coumaroyl shikimic acid displayed putative trends in the first stage, whereas, in the third stage, they were inconsistent with the putative results (**Figures S3**, **S4**). These irregular phenomena may be caused by other post-transcription regulating or modifications mechanisms.

## 4 Discussion

Early studies classified lncRNAs as transcriptional noise because of their low transcription levels and conservation. To date, lncRNAs are increasingly recognized as an important regulatory molecule in animals and plants. In plants, Numerous studies have proven lncRNAs’ key roles in many biological processes, including flower time (Heo and Sung, 2011), nutrition metabolism (Borah et al., 2018), root development (Chen et al., 2018a), abiotic stress (Gai et al., 2018), etc. In this study, we conducted lncRNAs sequencing for the flower of *M. candidum*. In total, 1,499 DElncRNAs were identified. Several lncRNAs with *cis*, trans, and antisense functions on mRNA were identified, and the lncRNAs-mediated ceRNA regulatory mechanism was verified. Our research proved that lncRNAs play a pivotal role in flower development and color control in *M. candidum* via direct or indirect ways.

The statistics databases of lncRNAs include CPC, CNCI, PFAM protein structure domain (PFAM), and coding potential assessment tool (CPAT), which will lead to significant discrepancies in the number of lncRNAs detected. For example, by using CPC and CNCI databases, Zhu et al. (2019a) identified 32,036 lncRNAs in the leaf and shoot of *Camellia sinensis*. Ye et al. (2019) identified 1,860, 3,342, 6,102, and 5,543 lncRNAs by CNCI, CPC, PFAM, and CPAT databases, respectively, in eight tissues of *Ginkgo biloba*, finally, they obtained 1,270 common lncRNAs. Our sequencing results showed that CPC2 and CNC, respectively, produced 4,508 and 6,567 lncRNAs. Finally, 3,955 common lncRNAs based on Venn analysis were identified. Our lncRNA number is more than Ye et al.’s research but far less than Zhu et al.’s research. Since lncRNAs lack codon regions, they are less conserved than protein-coding genes. They may possess a conserved motif but are not easily detected by the BLAST method (Kang and Liu, 2015). Numerous studies had reported that lncRNAs also possessed tissue-specific expression profiles (Ding et al., 2019; Zong et al., 2021). Pairwise comparisons between tissues and stages revealed different proportions of lncRNA isoforms and loci among different tissues or stages in *Fragaria vesca* (Kang and Liu, 2015). So, we concluded that these differences in detected lncRNA numbers in our research are normal and they may be caused by the different detected tissues and species. Further analysis showed that our lncRNAs data was moderate and sufficient to investigate their regulatory relationships involved.

The importance of lncRNAs in coloring and pigment formation has been demonstrated by several studies. For example, TCONS_01039552 and PONTK.3920.2 could regulate the expression of *F3H* in sea buckthorn fruit and *Solanum tuberosum*, respectively (Zhang et al., 2018; Bao et al., 2022). Our research identified two lncRNAs, lnc-MSTRG.10215.1 and lnc-MSTRG.11402.2, that can regulate *F3H* via ceRNA regulatory mechanism. (**Figure 6A**). In strawberry fruit, TRINITY_DN48515_c0_g3_i1 and TRINITY_DN1328_c0_g1_i1 could positively and negatively correlate with *CHI* and *CHS*, respectively (Lin et al., 2018); in *Solanum tuberosum* L, PONTK.2668.1 and PONTK.2668.15 could regulate *CHS* (Bao et al., 2022). In our research, we observed that Lnc-MSTRG.30526.3 and lnc-MSTRG.30533.1 could regulate *CHSJ* by both *cis* and antisense models (**Figures 3C, D**). Lnc-MSTRG.20470.1 could regulate *CHI1* through the antisense model (**Figure 3D**). Zhu et al. (2019a) found that LTCONS00054003 targets *4CL* by *cis* model in fresh leaf and shoot of *Camellia sinensis*. Similarly, our research found that lnc-MSTRG.28304.1 targeted *4CL3*, one member of *4CL*. In addition, lnc-MSTRG.28377.1 targeted on two *COMT1* members, *McCOMT1a* and *McCOMT1b*, and Lnc-MSTRG.19485.1 targeted *McMYB4* (**Figure 3C**). Previous studies demonstrated that *4CL3* displays a strong preference for 4-coumaric acid as substrate and is expressed at high levels in flowers but not in lignified organs suggesting that the primary function of *4CL3* is to provide activated 4-coumaric acid for the chalcone synthase (CHS) reaction that feeds the flavonoid-specific branch pathways in *Arabidopsis* (Ehlting et al., 1999; Kumar and Ellis, 2003). *AtMYB4* may affect pollen development by altering the flux of the phenylpropanoid pathway and pollen wall composition (Preston et al., 2004). COMT1 could catalyze the conversion of caffeic acid to ferulic acid and of 5-hydroxyferulic acid to sinapic acid (Gowri et al., 1991). COMT1 also methylates 5-hydroxyferuloyl CoA derivatives and flavonols with vicinal aromatic dihydroxy groups, such as quercetin (Fellenberg et al., 2012). In the antisense model, lncRNAs can pair with target genes, such as *McMYB1*, *McMYB17*, *McMADS4*, and *McMADS8* to regulate gene expression and control anthocyanin biosynthesis and pigment accumulation in floral organs. It was reported that a decrease of *MYB1* expression inhibits anthocyanin biosynthesis in bagged Chinese bayberry fruit, suggesting that *MYB1* may be involved in anthocyanin biosynthesis (Niu et al., 2010). In apples, the transcript level of *MYB17* was highly correlated with anthocyanin level, suggesting its role in pigment accumulation. The MADS-box motif has been identified in three different classes of genes in floral organs: A, B, and C. The genes of class A and C are responsible for the development of sepals and carpels, respectively. And the genes of classes B and C together control the formation of stamens (Kang et al., 1998). Additionally, we found some genes exist in both the ceRNA regulatory mechanism and the *cis* model, including *AHT1*, *UGT88B1*, and *CYP98A1*. These results indicated that lncRNAs are key factors to controll flower color formation and development through diverse mechanisms in *M. candidum*.

In the trans model of lncRNA regulation, lncRNAs are able to control gene expression from a distance by interacting with target genes at a different chromosomal locus. (Fukuda et al., 2019; Fu et al., 2020). Previous study demonstrated that DEGs regulated by lncRNAs in the trans model were significantly higher than DEGs regulated by *cis* and miRNA-mediated models, in which 413 lncRNAs were found to be capable of regulating 6060 genes in the trans model (Zong et al., 2021). Our GO and KEGG analysis results also showed a similar phenomenon. According to our results, a lot of lncRNAs have been shown to regulate protein-coding genes. In GO analysis, we found that the antisense model of lncRNAs could not be detected in the flavone biosynthesis process, or the alkaloid metabolic process; *cis* model of lncRNAs could not be detected in the tropane alkaloid biosynthetic process, and the alkaloid metabolic process. KEGG analysis results revealed that the antisense model of lncRNAs was missing in isoflavonoid biosynthesis pathways, folate biosynthesis pathways, flavone and flavonol biosynthesis pathways, as well as brassinosteroid biosynthesis pathways. The *cis* model of lncRNAs was not observed in the pathway for the synthesis of isoflavonoids and anthocyanins. In addition, the enriched gene numbers in the trans model were also significantly higher than in other acting models (**Figures 3A, B**). While numerous studies have demonstrated that these three acting models exist, researchers are unable to confirm their role directly due to the absence of related techniques. It is urgent to find a method to affirm the exact function of lncRNAs.

Tobacco injection assay is usually influenced by many factors such as ratio of GV3101 harboring different vectors, activity of the bacteria, injection volume of bacteria, and state of the tobacco leaves. These factors could influence experiment result directly. In our qRT-PCR assay of tobacco, we observed that the expression levels of *McUGT88F3* were higher in pCAMBIA2300-lnc-MSTRG.23785.1 + pCAMBIA2300-miR1220 + pCAMBIA1300-*McUGT88F3* combination than in pCAMBIA2300 + pCAMBIA1300-*McUGT88F3* combination. This phenomenon was not consistent with our expectation. In one hand, although we have controlled OD of bacteria to 0.7 - 1.0 and standardized the final OD to ~ 0.8 before injection, the exact states of the bacteria, such as viability of the bacteria and DNA quality in the bacteria, are still hard to keep consistent, which could affect the experiment results. Furthermore, the quality of tobacco leaves, such as the thickness, age, and texture, could affect the efficiency and volume of the injection, eventually causing different expression levels of target genes. Therefore, strict control of the experimental conditions is crucial for the accurate interpretation of the results. Another possible reason is that there may be highly conservated miRNAs in tobacco and *M*. *candium*. When injecting the exogenous target genes, the endogenous miRNAs of tobacco could also degrade these exogenous target genes, leading to the decrease of the expression level of them. The presence of lncRNA may contribute to the enhancement of *McUGT88F3* expression by protecting it from degradation by both endogenous and exogenous miRNAs, eventually leading to higher expression level of target genes in experiment group than in control group. Further studies are needed to confirm these hypotheses.

## 5 Conclusion

Collectively, the sole flower color of *M. candidum* greatly limits its application in the gardening industry, which is also a common problem for many garden plants. In our research, we identified specific lncRNAs that regulate flower development or color formation by comparing different stages of flower development of *M*. *candidum*. Plant development involves a complex set of physiological and biochemical reactions, such as splicing, methylation modification, histone modification at post- transcription or translation level, which could indirectly influence the expression patterns of functional genes. Since most current research focuses mainly on the direct regulation of functional genes or enzymes, some other key factors controlling flower color may be neglected. Despite lncRNAs being a relatively new classification of non-coding RNAs, they play various roles in plants and should be more widely studied in the future. Our research has proven the pivotal role of lncRNAs during flower development and color formation, offering a new avenue for regulating flower color at non-coding RNA levels in *M*. *candidum*.

## Data availability statement

The datasets presented in this study can be found in online repositories. The names of the repository/repositories and accession number(s) can be found below: NCBI BioProject accession number: PRJNA884434.

## Author contributions

LR, SD and YY designed this research; HL and WW collected the samples for this research; HL analysed, visualized the data, and wrote the draft; HL, BT, RL, XD and YL conducted the verification experiments; LR, SD and YY revised the paper. All authors contributed to the article and approved the submitted version.
