# Supplementary material for: Long non-coding RNA-mediated competing endogenous RNA regulatory network during flower development and color formation in Melastoma candidum
Source: Front Plant Sci. 2023 Jul 27;14:1215044. doi: 10.3389/fpls.2023.1215044 (PMC10415103; doi:10.3389/fpls.2023.1215044)
Supplement: Supplementary file 1 [file DataSheet_1.docx]

Supplementary Material

**Long non-coding RNA-mediated competing endogenous RNA regulatory network during flower development and color formation in** ***Melastoma candidum***

**Hui Li^1, 2^, Wei Wang^1^, Rui Liu^3^, Botong Tong^3,4^, Xinren Dai^3^, Yan Lu^5^, Yixun Yu^2, *^, Seping Dai^1, *^ Lin Ruan^1, *^**

**Correspondence:**Lin Ruan

alinche@126.com;

Seping Dai

daiseping@126.com;

Yixun Yu

yuyixun@scau.edu.cn

# Supplementary Figures

## Supplementary Figures


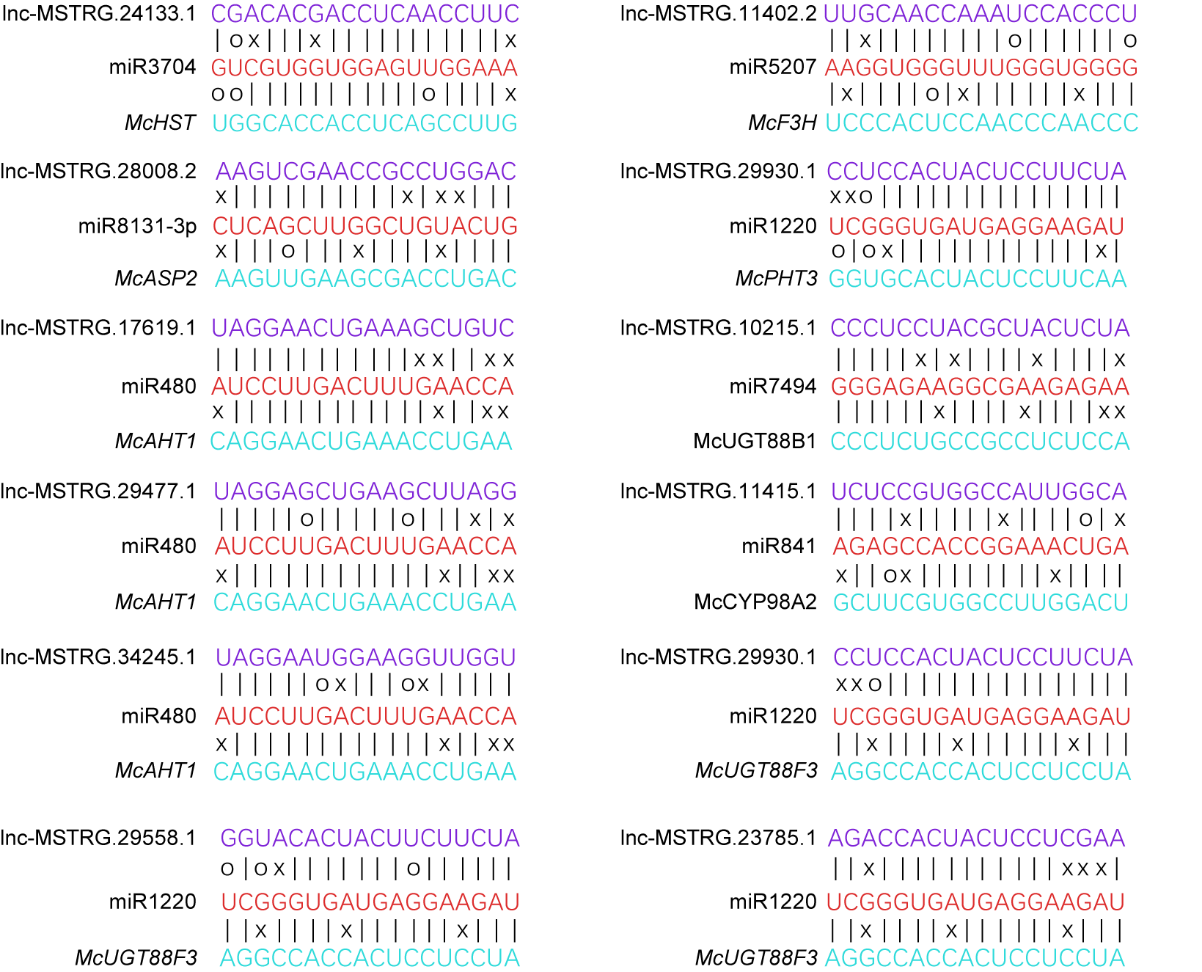


**Supplementary Figure 1.** Complementary bases between miRNAs and mRNA, circRNAs and miRNAs. "|" means complete complementary; "o" means complementary relations between G and U bases; "x" means no base pairing.


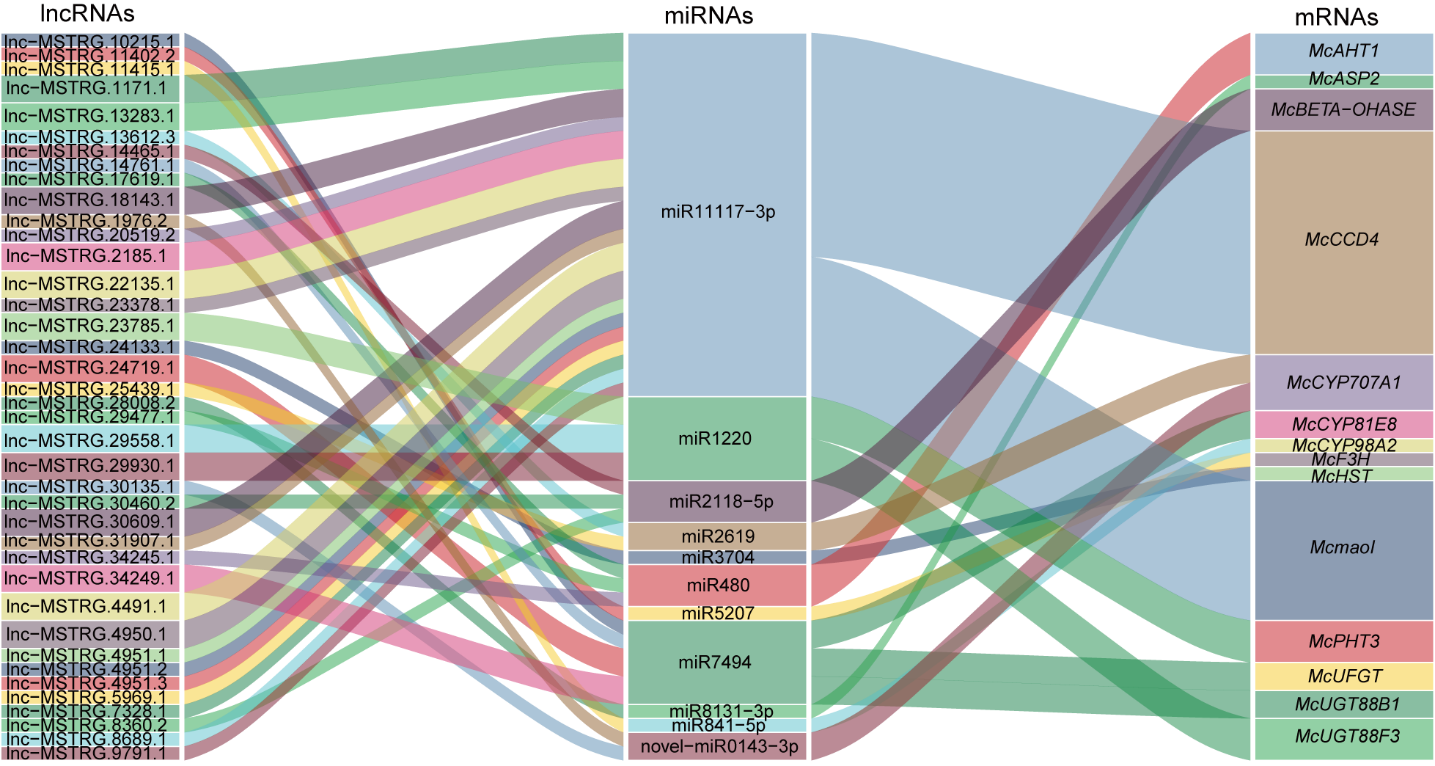


**Supplementary Figure 2.** ceRNA regulation relationship in flower development and flower color formation related pathway of *M*. *candidum*.


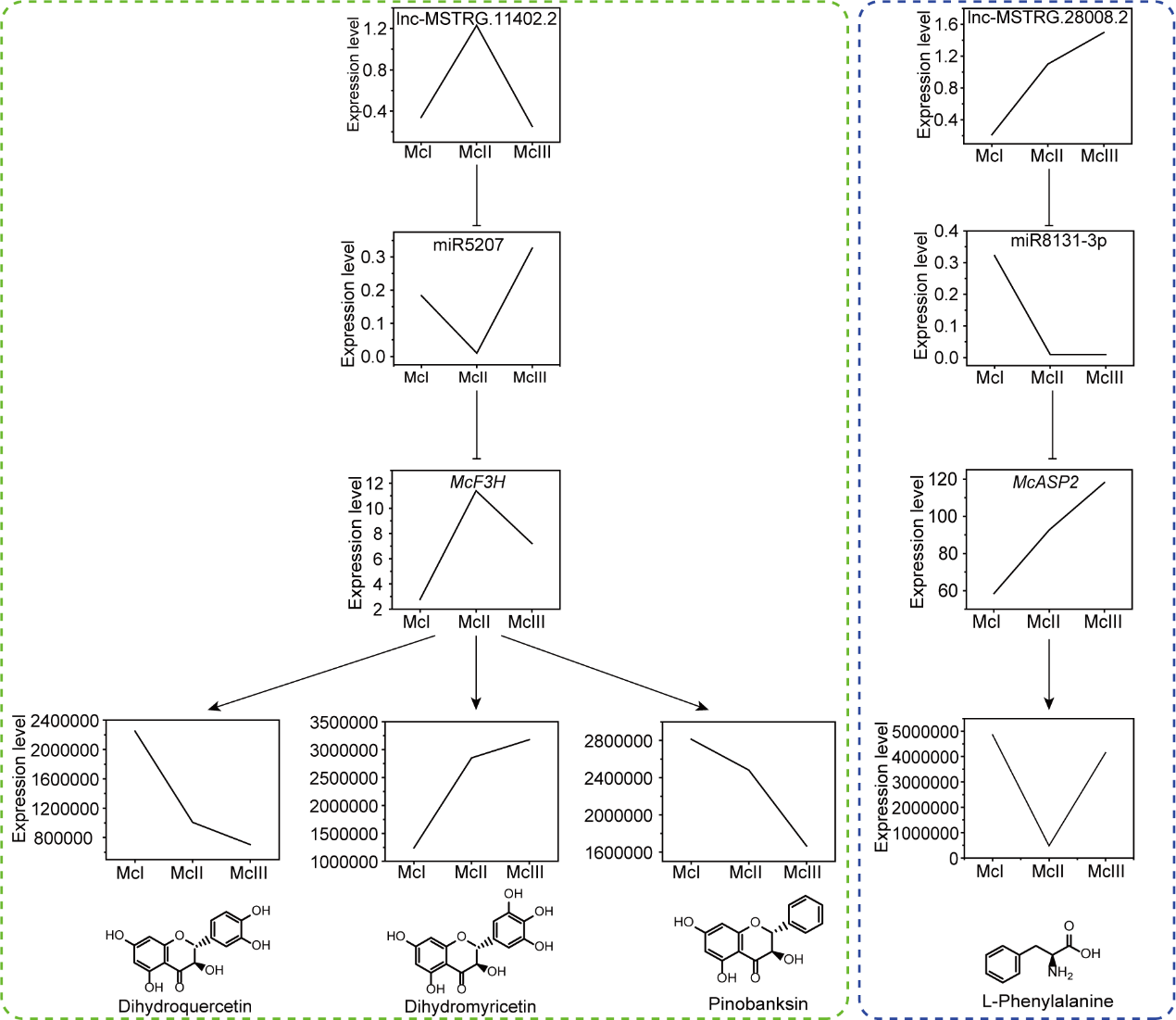


**Supplementary Figure 3.** Expression trends and regulation relationship of members within ceRNA regulatory network in flavonoids pathway**.**

**
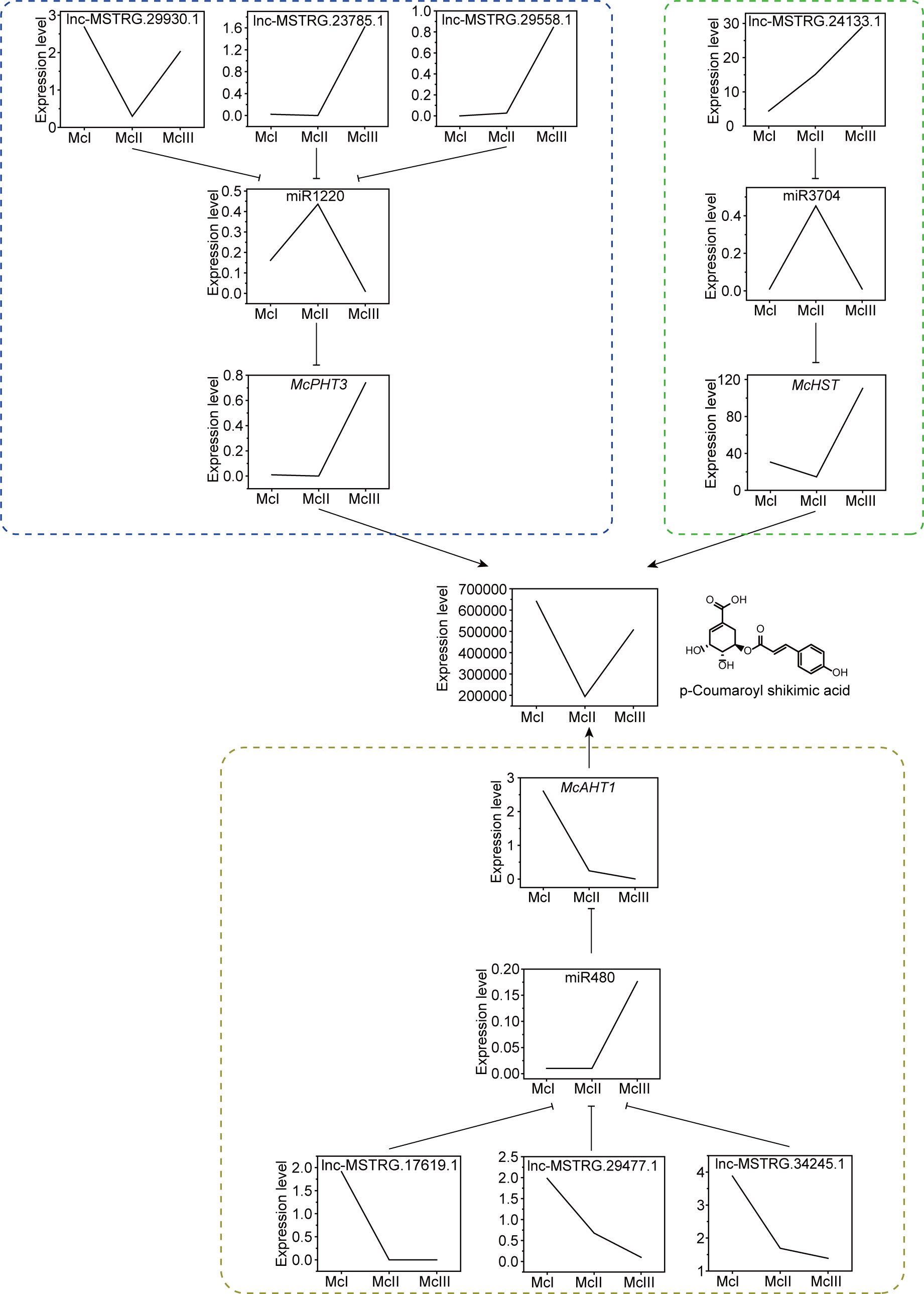
**

**Supplementary Figure 4.** Expression trends and regulation relationship of members within ceRNA regulatory network in flavone pathway.
